# Effectiveness of exercise intervention on improving fundamental motor skills in children with autism spectrum disorder: a systematic review and meta-analysis

**DOI:** 10.3389/fpsyt.2023.1132074

**Published:** 2023-06-12

**Authors:** Yu-Qin Ji, Hao Tian, Ze-Yu Zheng, Zhuo-Yan Ye, Qiang Ye

**Affiliations:** ^1^School of Sport and Health, Nanjing Sport Institute, Nanjing, Jiangsu, China; ^2^School of Physical Education and Humanities, Nanjing Sport Institute, Nanjing, Jiangsu, China; ^3^Department of Sport, Gdansk University of Physical Education and Sport, Gdansk, Poland; ^4^Nanjing Foreign Language School Xianlin Campus, Nanjing, Jiangsu, China

**Keywords:** autism spectrum disorder, children, exercise, fundamental motor skills, meta-analysis

## Abstract

**Background:**

Autism spectrum disorder (ASD) is a severe public health concern, and most of the children with ASD experience a substantial delay in FMS. This study aimed to investigate the effectiveness of exercise interventions in improving FMS in children with ASD, and provide evidence to support the scientific use of exercise interventions in practice.

**Methods:**

We searched seven online databases (PubMed, Scopus, Web of Science, Embase, EBSCO, Clinical Trials, and The Cochrane Library) from inception to May 20, 2022. We included randomized control trials of exercise interventions for FMS in children with ASD. The methodological quality of the included studies was assessed using the Physiotherapy Evidence Database Scale. Stata 14.0 software was used for meta-analysis, forest plotting, subgroup analysis, heterogeneity analysis, and meta-regression.

**Results:**

Thirteen studies underwent systematic review (541 participants), of which 10 underwent meta-analysis (297 participants). Overall, exercise interventions significantly improved overall FMS in children with ASD. Regarding the three categories of FMS, exercise interventions significantly improved LMS (SMD = 1.07; 95% CI 0.73 to 1.41, *p* < 0.001), OCS (SMD = 0.79; 95% CI 0.32 to 1.26, *p* = 0.001), and SS (SMD = 0.72; 95% CI 0.45 to 0.98, *p* < 0.0001).

**Conclusion:**

exercise interventions can effectively improve the FMS of children with ASD. The effects on LMS are considered as large effect sizes, while the effects on OCS and SS are considered as moderate effect sizes. These findings can inform clinical practice.

**Systematic review registration:**

https://inplasy.com/inplasy-2022-12-0013/.

## 1. Introduction

Autism spectrum disorder (ASD) is a complicated neurodevelopmental disorder. The estimated prevalence of ASD is ~1% of the current global population (1). According to the Diagnostic and Statistics Manual of Mental Disorders (DSM-5), individuals with ASD have two core symptoms: severe deficits in social communication behaviors and highly restrictive, repetitive behaviors (2). Previous studies have dominantly focused on these two key areas (3). Studies have also established that individuals with ASD suffer from other health problems or risks, including sleep disturbances (4), obesity (5), executive function deficits (6), physical inactivity (7), and motor dysfunctions (8). While higher levels of physical activity are beneficial for lifespan development and overall health of individuals. Exercise as purposed, structured and repeatedly physical activity can ameliorate health problems. Accumulating evidence has shown that exercise intervention improve a variety of typical impairments in children with ASD, such as social skills (9), stereotypic behaviors (10), and motor competence (11). And exercise intervention is potential for individuals with ASD to adapt their lives, which is engaging a growing area of research interest (12).

Recently, fundamental motor skills (FMS) have been widely regarded as an essential prerequisite for participation in physical activity. They are the essential building blocks for further, more complex motor competency and motor skills (13). FMS generally developed during childhood and subsequently refined into specific skills, including locomotor skills (LMS, e.g., running and hopping), object control skills (OCS, e.g., catching and throwing), and stability skills (SS, e.g., balancing and twisting) (13). Mastery of FMS is reported to contribute to children's physical (14), cognitive (15), and social development (16) and is considered a foundation for an active lifestyle (13, 17, 18). However, a couple of studies have mentioned that children with ASD experience a substantial delay in FMS compared to age-matched children who are typically developing (TD) (19, 20). Pan et al. (21) deployed the Test of Gross Motor Development-Second Edition (TGMD-2) to assess FMS performance in children aged 6–10 years with TD and ASD. They found that LMS and OCS scores of ASD were the lower than TD. Liu et al. (19) evaluated the FMS performance of 30 children with ASD and 30 age-matched children with TD using the Movement Assessment Battery for Children-2 (MABC-2). They found that the FMS performance in children with ASD was lower than that with TD. Although children with ASD generally have deficits in FMS, these deficits are not irreversible (12). Therefore, it is critical to promote FMS in the childhood of children with ASD (13).

With the rapidly increasing studies on exercise intervention to promote FMS in children with ASD, several reviews have begun to summarize the effects of exercise intervention. A systematic review examined the effects of motor and exercise interventions on motor function in children with ASD, and found that exercise interventions improve motor participation, activity, and body functional outcomes (22). Meanwhile, a meta-analysis examined the effects of various exercise-based interventions on gross motor skills in children with ASD. The results demonstrated that exercise interventions had a large effect on overall gross motor function (g = 0.99, *p* < 0.001) (11). Notably, this study didn't investigate the effect of exercise interventions on the categories of gross motor function. Recently, another systematic review first explored the effects of motor skills interventions on LMS, OCS and SS in children with ASD. Their results found that 86.3% of participants reported significant effects (23). Although studies have confirmed the beneficial effects of exercise interventions on motor competence in children with ASD, there are still some underlying questions. First, previous reviews focused on motor skills or gross motor skills rather than FMS. Secondly, few reviews conducted a meta-analysis on FMS and examined subsequently on LMS, OCS, and SS. Thirdly, most of the studies included in relevant reviews were none randomized controlled trials (RCTs) with low quality. Finally, no review has explored the impact of different FMS measurement tools (which assess different categories of FMS) on the intervention effect.

To the best of our knowledge, no meta-analysis has examined the effect of exercise intervention on FMS and its categories in children with ASD. As a result, to address these underlying issues in the literature and provide theoretical basis for formulating exercise prescriptions, this meta-analysis aims to synthesize published studies to quantify the effect sizes of exercise interventions on FMS and its categories in children with ASD.

## 2. Materials and methods

The presented study was accomplished in compliance with the Preferred Reporting Items for Systematic Review and Meta-Analysis (PRISMA) guidelines (24). Furthermore, it was registered in INPLASY (doi: 10.37766/inplasy2022.12.0013).

### 2.1. Data sources and searches

We searched seven online databases (PubMed, Scopus, Web of Science, Embase, EBSCO, Clinical Trials, and The Cochrane Library) from inception to May 20, 2022. Medical subject headings used were “autism spectrum disorder” AND “children” or “adolescents” AND “fundamental motor skills” AND “physical activity” or “exercise.” Meanwhile, we manually searched the included studies' reference lists, relevant systematic reviews and meta-analyses. In addition, a secondary search was conducted on November 5, 2022, to append the most recent literature. Supplementary Table 1 describes the detailed search strategy.

### 2.2. Study selection/inclusion criteria

Participants: Children mean age ≤ 18 years who were diagnosed with ASD by the Diagnostic and Statistical Manual of Mental Disorders (DSM-4, or DSM-5), the Autism Diagnostic Interview-Revised (ADI-R), the Autism Diagnostic Observation Schedule (ADOS-2), Gilliam Autism Rating Scale (GARS-2), or other standardized diagnosis criteria.

Interventions: Exercise interventions included structured or unstructured training, exercise, or physical activities.

Control conditions: Treatment as usual, waitlist, or a control group (not treated with any physical activity or exercise).

Outcome measures: The outcome measure was assessed by validated tools, such as the Bruininks-Oseretsky Test of Motor Proficiency, Second Edition (BOT-2), TGMD-2, or other standardized tools. Outcome indicators included quantitative data on FMS (LMS, OCS, SS), at least one used to calculate the summary effect size.

Study design: Only RCTs were eligible.

### 2.3. Exclusion criteria

Studies that meet the following criteria are excluded: the study subject's age was out of the limits; the study data could not be extracted; a publication that was a case study, review, or conference paper; the exercise intervention group contained confounding factors other than exercise, such as behavioral interventions, drugs etc.; studies not published in English.

### 2.4. Data extraction

After removal of duplicates, two researchers (JYQ and ZZY) independently screened titles, abstracts, and full-texts using Endnote X9 to make an initial assessment. A third authors (TH) was available for mediation throughout this process.

Data were extracted from the included studies according to a predefined protocol by two authors (JYQ and TH) and cross-checked for accuracy by a third author (YQ). The data extracted included: basic details of literature (first author name, year of publication, and country/region); participant characteristics (diagnosis, age range, sex, sample size); intervention components (type, session time, frequency, duration, measurement tools); major findings (LMS, OCS, SS).

### 2.5. Study quality

Study quality was scored by The Physiotherapy Evidence Database Scale (PEDro) (25). The PEDro is a well-proved scale for assessing the study quality of exercise intervention on children with ASD (26). The PEDro scale includes 11 rating criteria regarding eligibility, randomization, allocation, blinding (subjects and experimenter), intention-to-treat, between-group comparison and point measures. PEDro scale scores range from 0 to 10. Notably, one review stated that blinding might be unrealistic in exercise interventions (27). Thus, blinding was ignored due to the limitations of exercise interventions (28). Three levels ranked the quality of one study: low quality with a score <4, medium quality with a score of 4–5, and high quality with a score ≥6 (29). The quality of included studies were independently assessed by two authors based on PEDro, and a third author resolved any disagreements.

### 2.6. Data analysis

Stata 14.0 software (Stata, Texas, USA) was used for data analysis. The included data were continuous variables. The effect sizes (ESs) were calculated by Standardized mean difference (SMD) and 95% confidence intervals (CI). In the absence of sufficient data to conduct meta-analyses, the magnitude of ESs was calculated by Hedges' g (30). 0.2, 0.5, and 0.8 represents thresholds for small, medium, and large effects (31). Heterogeneity was determined with the *p*-value (threshold point of 0.1) and *I*^2^ statistic (25, 50, 75% representing thresholds for small, medium, and large ratios of inter-study heterogeneity) (32). We applied the fixed-effect model if no statistical heterogeneity was found across studies (*I*^2^ ≤ 50%, *p* > 0.1). Otherwise, the random-effects model was used (26).

Due to different frameworks among several measurement tools, the overall outcomes are hardly measured based on a consistent standard. Therefore, we analyzed the effect on the three sub-categories (LMS/OCS/SS) of the FMS (13). Considering that the ESs may be influenced by heterogeneity factors (FMS measurements, intervention type, intervention time, intervention frequency, intervention duration), Subgroup analyses were conducted. Furthermore, regression analyses were also performed for measurements, type, time, frequency, and duration. Since fewer than ten studies were included in each analysis, publication bias was not investigated. Sensitivity analysis was performed by excluding individual studies one by one using Stata 14.0 software.

### 2.7. Evidence certainty assessment

Two independent researchers assessed the quality of the evidence for each outcome using the Grading Recommendations to Assess Development and Evaluation System (GRADE), which is one of the international standards for quality of evidence and the classification of recommendation strength. The quality of the evidence can be classified to four levels: I (high); II (moderate); III (low); IV (very low) (33). Five factors can decrease the quality of the evidence: (1) risk of bias; (2) imprecision; (3) inconsistency; (4) indirectness; and (5) publication bias. Three factors can increase the quality of the evidence: (1) effect size; (2) dose-response gradient; and (3) control of confounding variables.

## 3. Results

### 3.1. Literature search results

**Figure 1:**
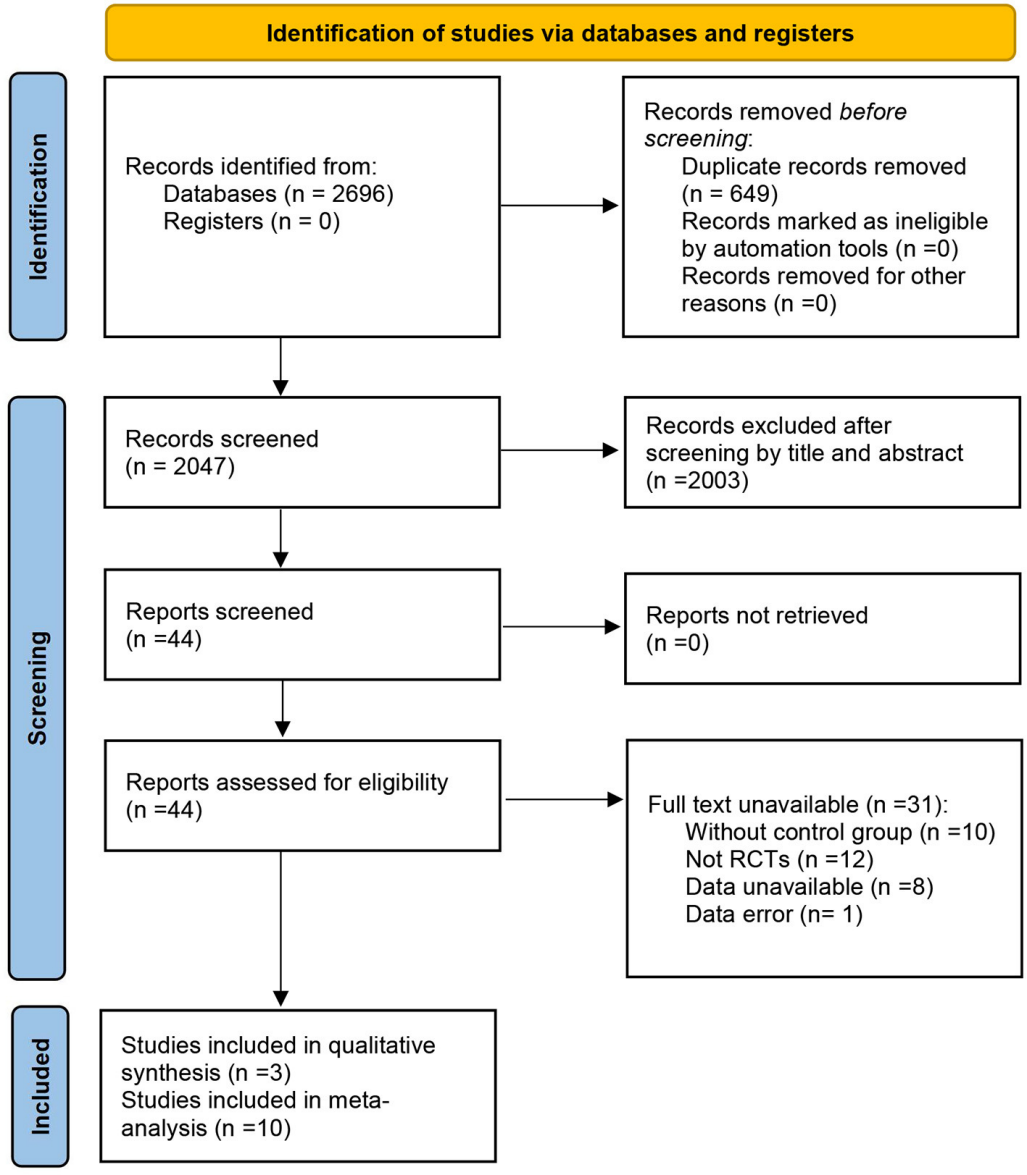
PRISMA flow diagram of the selection studies (37).

The initial search found 2,696 articles from seven databases. After removing 649 duplicated articles, 2,047 were screened by titles and abstracts. Subsequently, full-text screening was retrieved for 44 articles, of which 13 were eligible for inclusion. Finally, 13 articles were included in the systematic review, and ten were included in the meta-analysis. Three articles were excluded from the meta-analysis as they only reported the FMS total score (34–36). Figure 1 shows the screening process for the study and the reasons for exclusion (37).

### 3.2. Characteristics of included trials

Thirteen included RCTs studies listed in Table 1. In terms of years, ten studies were conducted within the past 5 years (34–36). In terms of the Country and region, seven studies were conducted in Iran (38–43) and Taiwan (44); the remains were from the USA (36), UK (35), Italy (34), India (45), Brazil (46), and Tunisia (47). A total of 541 participants were included, of which 298 were in the exercise intervention group, with a sample size range from 16 to 116. The age of the included sample ranged from 5 to 16, with 84.3% of the sample being male.

**Table 1:** Descriptive characteristics of included studies.

**Study, Country**	**Participant characteristics**		**Intervention characteristics**				**Outcome measured**	**Main findings**
	**Age range; sex-M%; Diagnosis methods**	**Sample size (IG/CG)**	**Type**	**Time (min)**	**Frequency (weekly)**	**Duration (week)**		
Ansari et al. (38)^b^; Iran	8~14; M-NR; DSM-5	30 (10/10/10)	Aquatic/Kata Training	60	2	10	Stork standing test Walking Heel to Toe Test	Static balance^aAquatic^ (*P* = 0.012) Dynamic balance^aAquatic^ (*P* = 0.001) Static balance^aKata^ (*P* = 0.001) Dynamic balance^aKata^ (*P* = 0.001)
Arabi et al. (39)^b^; Iran	6~12; M-76.7%; ADI-R/DSM-5	30 (15/15)	SPARK	60	3	10	TGMD-2	LMS^a^ (*P* = 0.001) OCS^a^ (*P* = 0.001) Total^a^ (*P* = 0.001)
Borgi et al. (34); Italy	6~12; M-NR; DSM-4/ICD-10	28 (15/13)	Horseback Riding	65	1	25	VABS	Total ^c^ (*p* > 0.05)
Dickinson et al. (35); UK	5~15; M-79%; NR	100 (50/50)	CB Training	15	3	16	Jump test Shuttle test Sit-ups Sit and reach test	Shuttle run^a^ (*p* < 0.001) Broad jump^c^ (*p* > 0.05) Sit-ups^c^ (*p* > 0.05) Flexibility^a^ (*p* < 0.05)
Gabriels et al. (36); USA	6~16; M-87%; ADOS-2	116 (58/58)	Horseback Riding	60	1	10	BOT-2	Total^c^ (*P* = 0.26)
Hassani et al. (40)^b^; Iran	8~11; M-66.7%; DSM-5	30 (10/11/9)	SPARK/ICPL	60	1	16	BOT-2	GMS^aSPARK^ (*P* = 0.005) Fine motor skills^cSPARK^ (*P* = 0.086) Total^aSPARK^ (*P* = 0.005) GMS^aICPL^ (*P* = 0.005) Fine motor skills^aICPL^ (*P* = 0.005) Total^aICPL^ (*P* = 0.005)
Najafabadi et al. (41)^b^; Iran	5~12; M-NR; DSM-4	26 (12/14)	SPARK	60	1	16	BOTMP	Static balance^a^ (*P* = 0.009) Dynamic balance^a^ (*P* = 0.001)
Pan et al. (44)^b^; Taiwan	8~10; M-NR; DSM-4	22 (11/11)	Table Tennis	70	3	12	BOT-2	Fine motor skills^c^ (*P* > 0.05) Manual coordination^a^ (*p* < 0.01) Body coordination^a^ (*p* < 0.01) Strength and agility^a^ (*p* < 0.01) Total a (*p* < 0.01)
Rafiei et al. (42)^b^; Iran	6~10; M-95%; ADOS-2	60 (20/20/20)	SPARK/Kinect	35	3	8	MABC-2	Manual dexterity^cSPARK^ (*p* > 0.05) ACS^aSPARK^ (*p* < 0.05) Balance skills^cSPARK^ (*p* > 0.05) Manual dexterity^cKinect^ (*p* > 0.05) ACS^cKinect^ (*p* > 0.05) Balance skills^cKinect^ (*p* > 0.05)
Sarabzadeh et al. (43)^b^; Iran	6~12; M-77.7%; GARS-2	18 (9/9)	Tai Chi Chuan	60	3	6	MABC-2	Manual dexterity^c^ (*P* = 0.95) Ball skills^a^ (*P* < 0.001) Balance skills^a^ (*P* < 0.001) Total^a^ (*P* < 0.001)
Marzouki et al. (47)^b^; Tunisia	6~7; M-90.9%; DSM-5	22 (8/8/6)	TB/GB aquatic training	50	2	8	TGMD-2	LMS^aTB^ (*P* < 0.05) OCS^aTB^ (*P* < 0.05) LMS ^aGB^ (P < 0.05) OCS^aGB^ (*P* < 0.05)
Shanker et al. (45)^b^; India	5~15; M-81.3%; clinical	43 (23/20)	Yoga	45	1	12	BOT-2	Fine manual coordination^c^ (*p* = 0.536) Manual coordination^a^ (*p* = 0.019) Body coordination^a^ (*p* = 0.01) Strength and agility^c^ (*p* = 0.158) Total^a^ (*p* = 0.05)
Haghighi et al. (46)^b^; Brazil	6~10; M-56.3%; GARS-2	16 (8/8)	Combined physical training	60	3	8	The stork test on one foot	balance^a^ (*p* = 0.019)

ACS, Aiming and catching skills; ADI-R, Autism Diagnostic Interview-Revised; ADOS-2, Autism Diagnostic Observation Schedule (Second Edition); BOTMP, Bruininks-Oseretsky Test of Motor Proficiency; BOT-2, Bruininks-Oseretsky Test of Motor Proficiency (Second Edition); CARS, Childhood Autism Rating Scale; CB, computer-based; CG, control group; DSM, Diagnostic and Statistical Manual of Mental Disorders; GARS, Gilliam Autism Rating Scale; GB, game-based; GMS, gross motor skills; IG, intervention group; ICPL, I Can have a physical literacy; ICD c, M, male; MABC-2, Movement Assessment Battery for Children (Second Edition); min, minutes; NR, not reported; SPARK, Sports-Play and Active Recreation for Kids; TB, technical-based; TGMD-2, Test of Gross Motor Development (Second Edition); VABS, Vineland Adaptive Behavior Scales. ^a^Significant statistical improvement. ^b^Studies included in the meta-analysis. ^c^No statistically significant change.

Eleven studies reported the ASD diagnosis confirmed using the formal diagnostic criteria. Seven studies (34, 38–41, 44, 47) used the DSM-4 or DSM-5, often the gold standard diagnostic tool for psychologists (33). Additionally, two studies (36, 42) used ADOS-2, two (43, 46) used GARS-2, whereas two studies (35, 45) did not describe the diagnostic process. Moreover, six studies assessed autism severity: four (38, 41, 43, 46) used GARS-2, one (47) used CARS-2, and one (45) used Autism Treatment Evaluation Checklist (ATEC). The other seven studies did not mention autism severity.

The intervention type involved four approaches: game (e.g., Kinect tennis, SPARK), motor skill (e.g., tennis, yoga, tai chi, karate), horseback riding, and aquatic training intervention. The intervention frequency ranged from 1 to 3 times/week, with each session ranging from 15 to 70 min and duration ranging from 6 to 25 weeks. The effects of exercise interventions on FMS were measured by 13 studies using diverse tools, in which six studies assessed LMS, six examined OCS, and seven (38, 41–46) targeted SS. Two studies (39, 47) deployed TGMD-2 to assess LMS and OCS; five studies (36, 40, 41, 44, 45) assessed FMS by BOTMP or BOT-2, and two studies (42, 43) used MABC-2. Moreover, four general measures (Walking Heel to Toe Test, shuttle test, et. al.) were used to assess FMS in children with ASD (34, 35, 38, 46).

### 3.3. Study quality

The PEDro scores of included studies ranged from 5 to 7, indicating the study quality from moderate to high (moderate = 23%, high = 77%). All included studies had clear recruitment criteria and were similar at baseline. However, few studies have been conducted on concealed allocation and blinding. The final PEDro scores are presented in Table 2. In addition, sensitivity analysis showed that the combined results changed insignificantly, demonstrating that the meta results were relatively stable, as shown in the Supplementary Figures 1–3.

**Table 2:** Methodological quality assessment of included studies.

**Study (country)**	**EC**	**RA**	**CA**	**SAB**	**SB**	**TB**	**AB**	**DR**	**ITA**	**BC**	**PM**	**TS**	**OSQ**
Ansari et al. (38)^a^; Iran	1	1	0	1	0	0	0	1	1	1	1	6	High
Arabi et al. (39)^a^; Iran	1	1	0	1	0	0	0	1	1	1	1	6	High
Borgi et al. (34); Italy	1	0	0	1	0	0	0	1	1	1	1	5	Moderate
Dickinson et al. (35); UK	1	1	0	1	0	0	0	1	0	1	1	5	Moderate
Gabriels et al. (36); USA	1	1	0	1	0	0	1	1	0	1	1	6	High
Hassani et al. (40)^a^; Iran	1	0	1	1	0	0	0	1	1	1	1	6	High
Najafabadi et al. (41)^a^; Iran	1	1	0	1	0	0	1	1	1	1	1	7	High
Pan et al.; (44)^a^; Taiwan	1	1	0	1	1	0	0	1	1	1	1	7	High
Rafiei et al.; (42)^a^; Iran	1	1	1	1	0	0	0	1	1	1	1	7	High
Sarabzadeh et al.; (43)^a^; Iran	1	1	0	1	0	0	0	1	1	1	1	6	High
Marzouki et al.; (47)^a^; Tunisia	1	1	0	1	0	0	0	0	1	1	1	5	Moderate
Shanker et al.; (45)^a^; India	1	1	0	1	0	0	0	1	1	1	1	6	High
Haghighi et al.; (46)^a^; Brazil	1	1	0	1	0	0	0	1	1	1	1	6	High

Yes = 1, no = 0. AB, assessor-blinded; BC, between-group comparison; CA, concealed allocation; DR, dropout rate; EC, eligibility criteria; ITA, intention-to-treat analysis; OSQ, overall study quality; PM, points measures; RA, random allocation; SAB, similar at baseline; SB, subject blinded; TB, therapist blinded; TS, total score. ^a^Studies included in the meta-analysis.

## 4. Meta-analysis results

Among the 13 studies, 10 RCTs investigated exercise intervention effects on FMS in children with ASD and were included in the meta-analysis. Due to the different FMS measurement tools, the content included in the total FMS score is inconsistent. Moreover, the overall improvement effect on the FMS of children with ASD is hard to calculate. Therefore, we analyzed the effect on the three lower categories of the FMS (LMS/OCS/SS). In addition, four studies included two intervention groups, which were distinguished in the meta-analysis using a and b.

**Figure 2:**
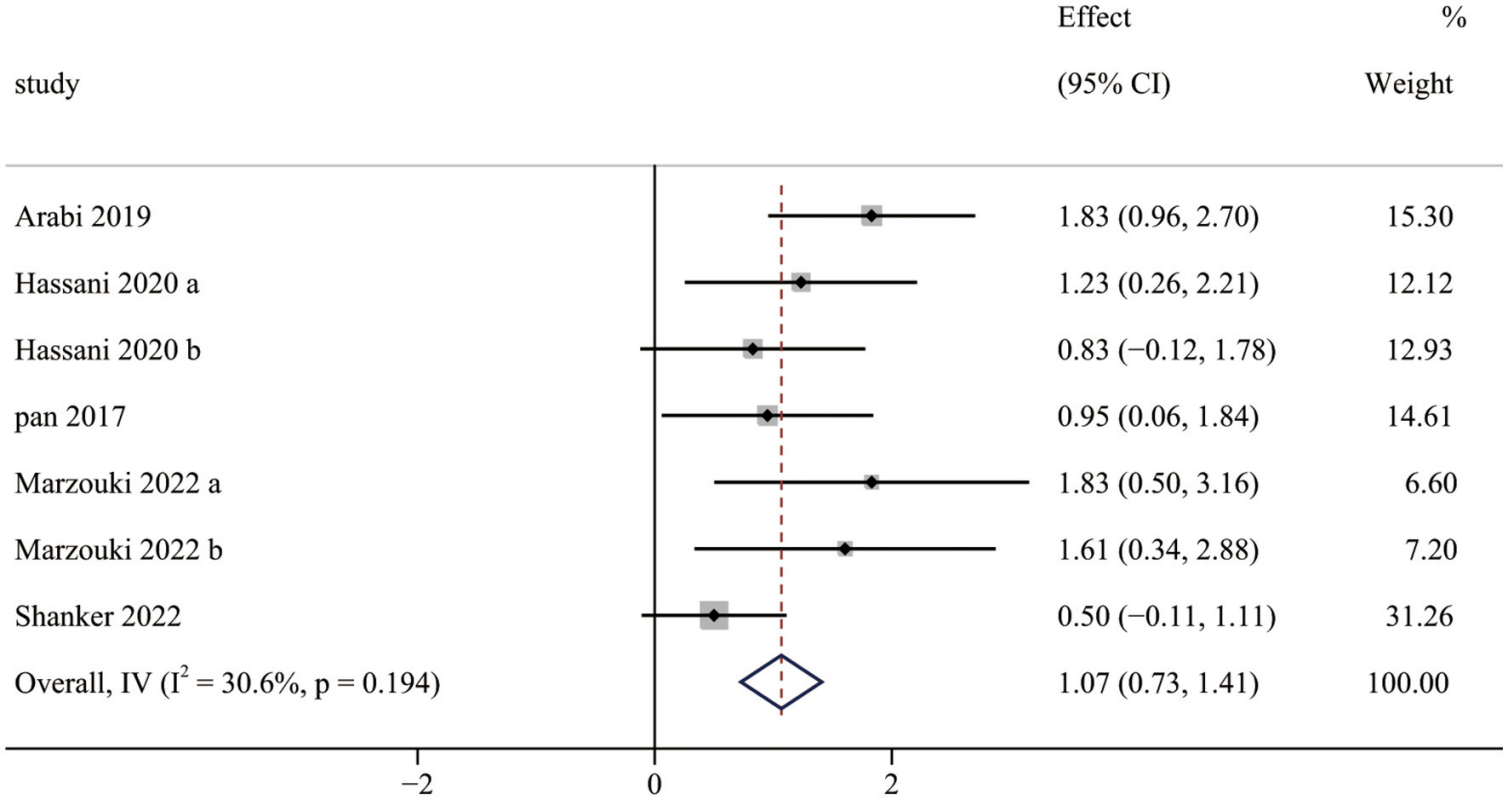
The effect of exercise interventions on LMS.

Five studies (seven pairwise comparisons) investigated the effect of exercise interventions on LMS. The combined results showed that exercise interventions significantly improved the LMS of children with ASD (SMD = 1.07; 95% CI 0.73 to 1.41; *p* < 0.001) (Figure 2). The SMDs of exercise intervention were considered high ESs.

**Figure 3:**
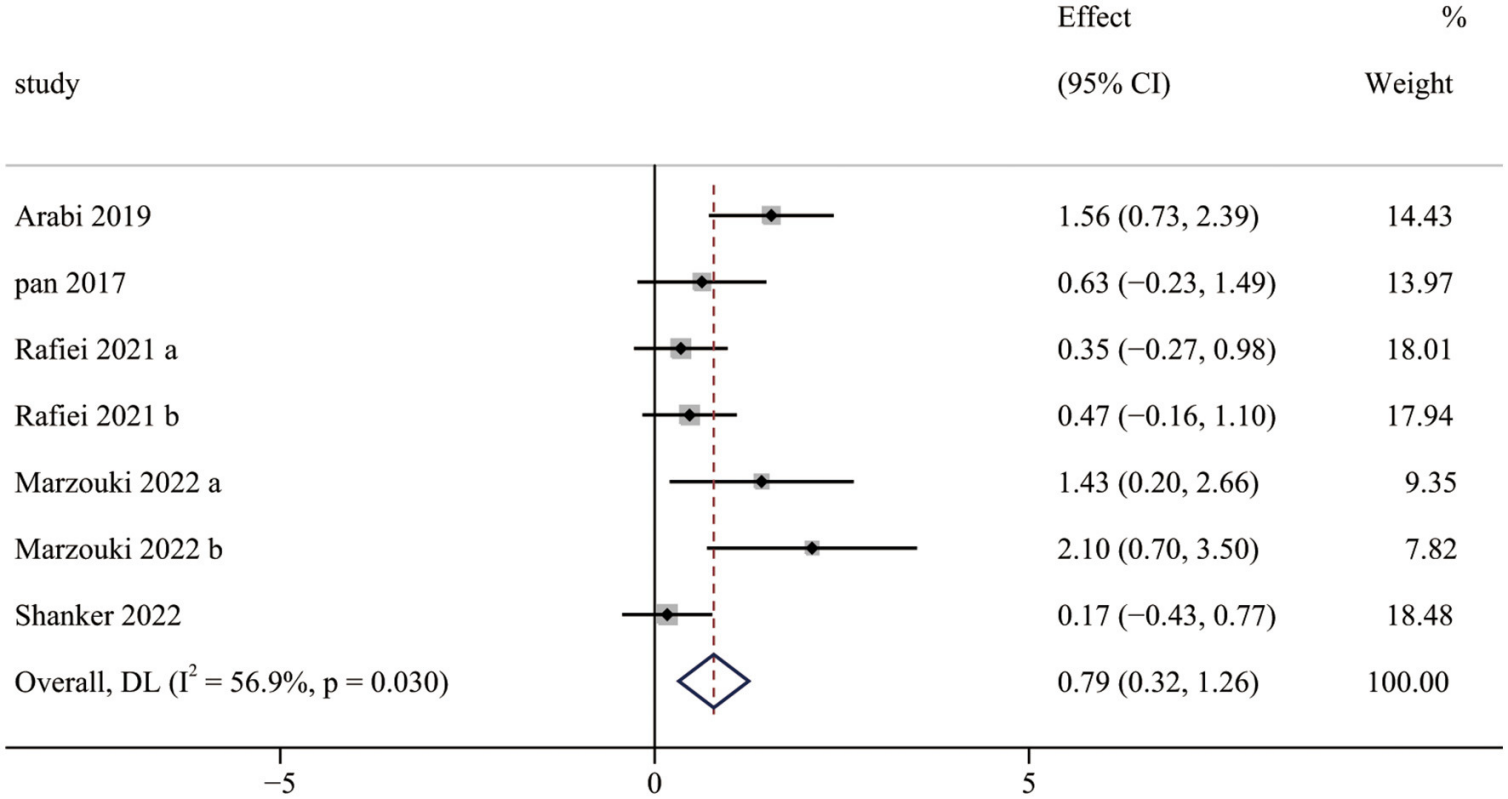
The effect of exercise interventions on OCS.

Five studies (7 pairwise comparisons) investigated the effect of exercise interventions on OCS. The combined results showed that exercise intervention significantly improved the OCS of children with ASD (SMD = 0.79; 95% CI 0.32 to 1.26; *p* = 0.001) (Figure 3). The SMDs of exercise intervention were considered moderate ESs.

**Figure 4:**
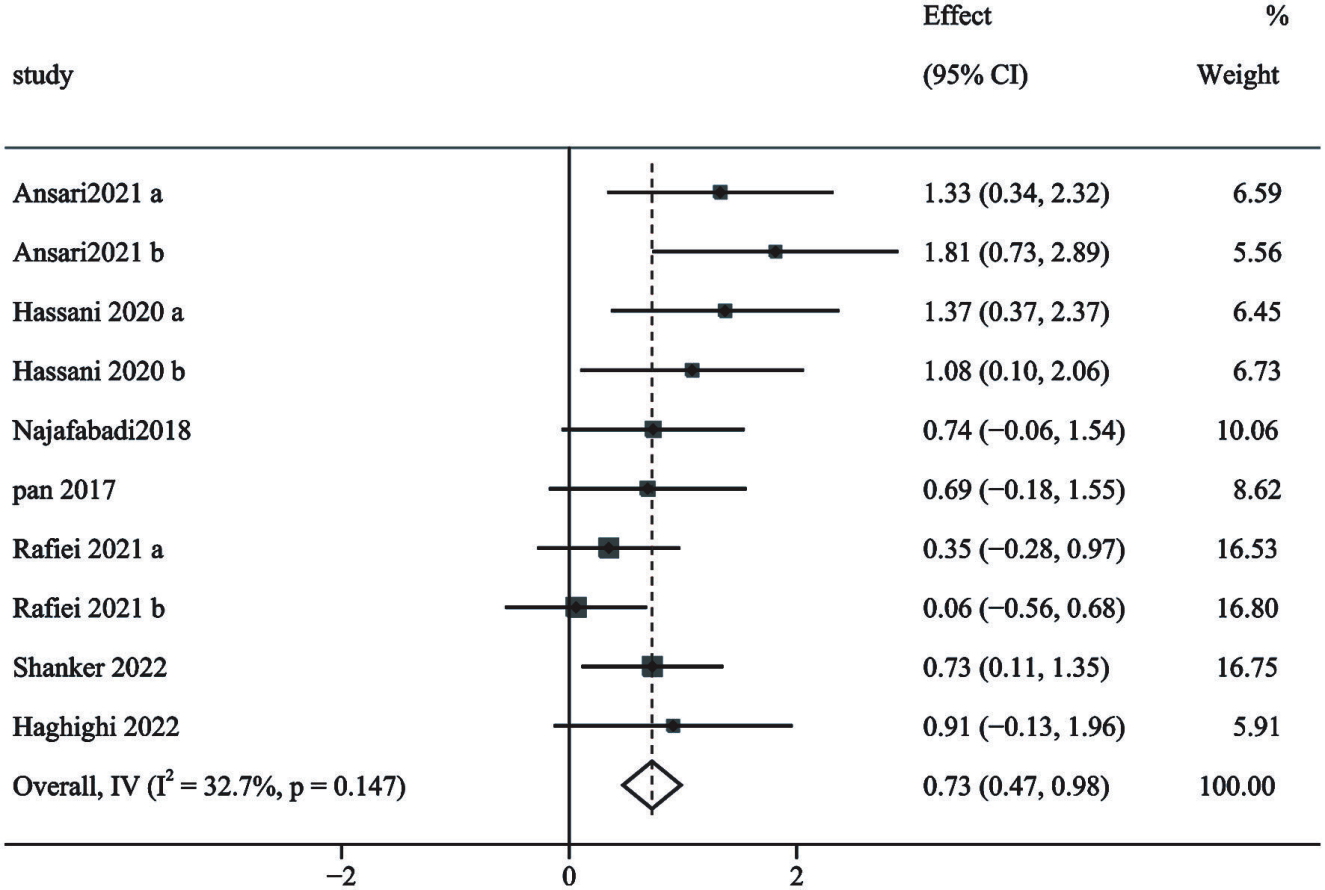
The effect of exercise interventions on SS.

Seven studies (10 pairwise comparisons) examined the effect of exercise interventions on SS. The combined results showed that exercise intervention significantly improved the SS of children with ASD (SMD = 0.73; 95% CI 0.47 to 0.98; *p* < 0.0001) (Figure 4). The SMDs of exercise intervention were considered moderate ESs.

### 4.1. GRADE quality evaluation

According to the criteria of GRADE, this study evaluated the certainty of the evidence regarding the significant improvement of exercise interventions on the categories (LMS, OCS, and SS) of FMS in children with ASD. Specifically, exercise interventions exhibited moderate-quality evidence for OCS and low-quality evidence for LMS and SS, as shown in the Supplementary Table 2.

### 4.2. Subgroup analysis

For exercise intervention, the variables of exercise intervention (type, duration, and frequency) and outcome measures are likely to influence children with ASD in their LMS, OCS, and SS. A moderator analysis was conducted using the corresponding model to investigate potential sources of variance.

#### 4.2.1. Locomotor skills

The subgroup analysis showed that measurement tools significantly moderated the effect of exercise interventions on LMS (Q = 6.77, *p* = 0.009). The ESs of the TGMD results (SMD = 1.78; 95% CI 1.14 to 2.40; *p* < 0.001) were significantly higher than that of the BOT results (SMD = 0.78; 95% CI 0.37 to 1.18; *p* < 0.001). Furthermore, the exercise intervention groups with 1–3 times/weeks were included in our current meta-analysis, indicating a statistically significant difference in the ESs (Q = 6.96; *p* = 0.031). The 2 times/week intervention had significant improvement in LMS (SMD = 1.78; 95% CI 1.14 to 2.40; *p* < 0.001) compared with 1 time/week (SMD = 0.73; 95% CI 0.28 to 1.19; *p* = 0.002) and 3 times/weeks (SMD = 0.95; 95% CI 0.06 to 1.84; *p* = 0.036). Similarly, the duration of intervention produced a statistically significant difference between the two subgroups (Q = 6.77, *p* = 0.009). The ESs for duration <12 weeks (SMD = 1.78; 95% CI 1.14 to 2.40; *p* < 0.001) was larger than for duration ≥ 12 weeks (SMD = 0.78; 95% CI 0.37 to 1.18; *p* < 0.001). Moreover, there were no statistical differences in the type (Q = 5.54, *p* = 0.063), and session time (Q = 1.04, *p* = 0.307) (Table 3).

**Table 3:** Subgroup analysis of LMS.

**Moderator**	***N***	**SMD**	**95% Conf. interval**	**Heterogeneity test results**		**Test for between-group heterogeneity**	
				***I^2^***	***P***	**Q-value**	***P*-value**
**Measurement** ^**^							
TGMD	3	1.78	1.14, 2.41	0%	0.956	6.77	0.009
BOT	4	0.78	0.37, 1.18	0%	0.619		
**Type**							
Game	3	1.33	0.79, 1.87	16.1%	0.304	5.54	0.063
Motor skill	2	0.65	0.14, 1.15	0%	0.414		
Aquatic training	2	1.71	0.80, 2.63	0%	0.811		
**Duration** ^**^							
≥12 weeks	4	0.78	0.37, 1.18	0%	0.619	6.77	0.009
< 12 weeks	3	1.78	1.14, 2.40	0%	0.956		
**Frequency** ^*^							
1 times/week	3	0.73	0.28, 1.19	0%	0.449	6.96	0.031
2 times/week	3	1.78	1.14, 2.40	0%	0.956		
3 times/week	1	0.95	0.06, 1.84	NR	NR		
**Exercise session time**							
≥60 min	4	1.23	0.77, 1.69	0%	0.409	1.04	0.307
< 60 min	3	0.87	0.37, 1.38	57.5%	0.095		

^*^Shows that the data differ. ^*^p < 0.05, ^**^p < 0.01.

#### 4.2.2. Object control skills

The subgroup analysis showed that measurement tools significantly moderated the effect of exercise interventions on OCS (Q = 12.5, *p* = 0.002). The ESs of the TGMD results (SMD = 1.63; 95% CI 1.01 to 2.25; *p* < 0.001) was higher than that of the BOT (SMD = 0.32; 95% CI−0.17 to 0.81; *p* = 0.202) and MABC-2 results (SMD = 0.41; 95% CI−0.04 to 0.85; *p* = 0.007). In terms of the type, a statistically significant ESs was found (Q = 6.95, *p* = 0.031) in the games (SMD = 0.74; 95% CI 0.06 to 1.41, *p* = 0.032), motor skill (SMD = 0.32; 95% CI−0.17 to 0.81; *p* = 0.20) and aquatic training (SMD = 1.72; 95% CI 0.80 to 2.65; *p* < 0.001). Notably, frequency significantly moderated the effect of exercise interventions on OCS (Q = 7.67, *p* = 0.022). Results revealed that the ESs for children with ASD engaged in a moderate-frequency (2 times/weeks) (SMD = 1.72; 95% CI 0.80 to 2.65; *p* < 0.001) was greater than those of in low-frequency (1 time/weeks) (SMD = 0.17; 95% CI−0.43 to 0.77; *p* = 0.581) or high-frequency (3 times/weeks) (SMD = 0.70; 95% CI 0.20 to 1.20; *p* = 0.006). By contrast, there were no statistical differences in duration (Q = 2.95, *p* = 0.086) and session time (Q = 0.71, *p* = 0.398) (Table 4).

**Table 4:** Subgroup analysis of OCS.

**Moderator**	***N***	**SMD**	**95% Conf. interval**	**Heterogeneity test results**		**Test for between-group heterogeneity**	
				***I^2^***	***P***	**Q-value**	***P*-value**
**Measurement** ^**^							
TGMD	3	1.63	1.01, 2.25	0	0.753	12.5	0.002
BOT	2	0.32	−0.17, 0.81	0	0.388		
MABC-2	2	0.41	−0.04, 0.85	0	0.797		
**Type** ^*^							
Game	3	0.74	0.06, 1.41	65.4	0.055	6.95	0.031
Motor skill	2	0.32	−0.17, 0.81	0	0.388		
Aquatic training	2	1.72	0.80, 2.65	0	0.479		
**Duration**							
≥12 weeks	2	0.32	−0.17, 0.81	0	0.388	2.95	0.086
< 12 weeks	5	1.02	0.39, 1.65	62.2	0.032		
**Frequency** ^*^							
1 times/week	1	0.17	−0.43, 0.77	NR	NR	7.67	0.022
2 times/week	2	1.72	0.80, 2.65	0	0.479		
3 times/week	4	0.70	0.20, 1.20	48.2	0.122		
**Exercise session time**							
≥60 min	2	1.10	0.19, 2.01	56.5	0.129	0.71	0.398
< 60 min	5	0.65	0.12, 1.17	53.6	0.071		

^*^Shows that the data differ. ^*^p < 0.05, ^**^p < 0.01.

#### 4.2.3. Stability skills

The subgroup analysis showed that measurement tools significantly moderated the effect of exercise intervention on SS (Q = 9.99, *p* = 0.007). The ESs of the general test results (SMD = 1.34; 95% CI 0.74 to 1.94; *p* < 0.001) was higher than that of the BOT (SMD = 0.86; 95% CI 0.49 to 1.22; *p* < 0.001) and TGMD results (SMD = 0.20; 95% CI−0.24 to 0.64; *p* = 0.366). For duration, the long-term (≥12 weeks) (SMD = 0.86; 95% CI 0.49 to 1.22; *p* < 0.001) had a significant improvement in SS compared with the short-term (<12 weeks) (SMD = 0.60; 95% CI 0.25 to 0.96; *p* = 0.001). Moreover, frequency significantly moderated the effect of exercise on OCS (Q = 9.01, *p* = 0.011). Results revealed that the ESs for children with ASD engaged in a moderate-frequency (2 times/weeks) (SMD = 1.55; 95% CI 0.82 to 2.28; *p* < 0.001) was greater than those of in low-frequency (1 time/weeks) (SMD = 0.89; 95% CI 0.49 to 1.30; *p* < 0.001) or high-frequency (3 times/weeks) (SMD = 0.38; 95% CI 0.01 to 0.75; *p* = 0.044). In addition, there were no statistical differences in type (Q = 3.14, *p* = 0.182) and duration (Q = 0.97, *p* = 0.326) (Table 5).

**Table 5:** Subgroup analysis of SS.

**Moderator**	***N***	**SMD**	**95% Conf. interval**	**Heterogeneity test results**		**Test for between-group heterogeneity**	
				***I^2^***	***P***	**Q-value**	***P*-value**
**Measurement** ^**^							
MABC-2	2	0.20	−0.24, 0.64	0%	0.524	9.99	0.007
BOT	5	0.86	0.49, 1.22	0%	0.813		
General	3	1.34	0.74, 1.94	0%	0.502		
**Type**							
Game	5	0.54	0.20, 0.87	40.3%	0.153	3.14	0.182
Motor skill	4	0.91	0.50, 1.33	8%	0.353		
Aquatic training	1	1.33	0.34, 2.32	NR	NR		
**Duration**							
≥12 weeks	5	0.86	0.49, 1.22	0%	0.81	0.97	0.326
< 12 weeks	5	0.60	0.25, 0.96	63%	0.029		
**Frequency** ^*^							
1 times/week	4	0.89	0.49, 1.30	0%	0.701	9.01	0.011
2 times/week	2	1.55	0.82, 2.28	0%	0.517		
3 times/week	2	0.38	0.01, 0.75	0%	0.472		
**Exercise session time** ^**^							
≥60 min	7	1.08	0.72, 1.43	0%	0.688	7.2	0.007
< 60 min	3	0.38	0.02, 0.74	11.3%	0.324		

^*^Shows that the data differ. ^*^p < 0.05, ^**^p < 0.01.

### 4.3. Meta-regression analysis

In order to investigate the exercise intervention effects on LMS, OCS, and SS, a meta-regression analysis was conducted to identify variables that might influence the effect of the intervention. Results indicated that measurement (β = −0.9962322; *p* = 0.048; CI−1.98 to−0.01) and duration (β = −0.9962322; *p* = 0.048; CI 0.01 to 1.98) significantly influenced LMS (Supplementary Table 3). Regarding the exercise intervention effects on OCS, all variables did not influence the ESs (Supplementary Table 4). In addition, results revealed that measurement (β = 0.581979; *p* = 0.014; CI 0.16 to 1.01) and exercise session time (β = −0.6958256; *p* = 0.028; CI−1.29 to−0.10) had a significant influence on SS, with higher session time leading to a more remarkable improvement in SS (Supplementary Table 5).

## 5. Discussion

To the best of our knowledge, the present study is the first systematic review and meta-analysis examining the effects of exercise interventions on FMS in children with ASD. The results demonstrated that exercise intervention has a moderate to high effect on three categories of FMS. Available evidence suggests that exercise intervention can significantly improve LMS (SMD = 1.07), OCS (SMD = 0.79), and SS (SMD = 0.73) compared with controls receiving no intervention.

### 5.1. Measurement

This study found a statistically significant difference between different FMS measurement tools. Every measurement tool has dominated function. For example, the TGMD-2 mainly assesses LMS (running, galloping, hopping, etc.) and OCS (two-hand strike, two-hand catch, kick, etc.) (48). The BOT-2 includes eight subtests (balance, upper limb coordination, strength, running speed and agility, etc.) that measure the LMS, OCS, and SS (49). In the included studies, FMS measurement in children with ASD involved various standardized tools, including TGMD-2, BOT-2, and MABC-2. By comparing their differences, the reasons for heterogeneity can be identified.

The present study found that TGMD-2 is better than BOT-2 and MABC-2. There are two general types of FMS measurement tools: product-oriented and process-oriented. Product-oriented tools measure the outcome of a motor skill performance, such as speed, distance, or time, also known as quantitative evaluation (50). In contrast, process-oriented tools mainly measure specific motor skill performance and completion process, also known as qualitative evaluation (50). Studies show that process- and product-oriented tools assess different aspects and do not equally evaluate intervention efficacy (51). In this study, the TGMD-2 is a process-oriented tool, while the MABC-2 and the BOT-2 are product-oriented tools, which explains the significant differences between the effects of separate measurement tools.

### 5.2. Locomotor skills

The meta-analysis demonstrates that exercise intervention has a large positive effect on LMS (SMD = 1.07), which is similar to previous results (9, 52). LMS is the ability of an individual to move within a spatial location, which was measured primarily through the TGMD and BOT. The possible reason is that specifically-designed and structured exercise interventions provide sufficient opportunities for children to perform the correct movement patterns to improve FMS (53). Foulkes et al. (54) implemented a 6-week active play (AP) intervention program in 162 preschool children aged 3–5 years and found no significant improvement in LMS and OCS. Moghaddaszadeh et al. (55) used a 7-week guided AP intervention program in 52 school-aged children aged 5–7 years, and found that LMS improved more significantly compared to the AP intervention group. It indicates that structured, guided exercise interventions improve the LMS of children with ASD rather than just giving children the opportunity to play freely.

Furthermore, our meta-analysis found moderate and significant heterogeneity between studies (*I*^2^ = 30.6%). The subgroup analysis revealed that a duration of <12 weeks had significantly higher effects than those of ≥12 weeks. This finding is in contrast to previous studies (11). Case et al. investigated the effect of different intervention approaches on gross motor outcomes of children with ASD and found that a total intervention time of ≥16 h was significantly higher than of <16 h. The possible reason is that the outcome measurement tools used for the studies (<12 weeks) were both TGMD, whereas the studies (≥12 weeks) were all evaluated by BOT, in which TGMD is a qualitative measurement tool, while BOT is a quantitative measurement tool. It hardly compared the results between the two types of measurement tools. Furthermore, the effects of interventions measured by TGMD were significantly higher than those measured by BOT. Notably, the effects of intervention with a moderate frequency (2 times/week) were significantly higher than those with low frequency (1 time/week) or high frequency (3 times/week). The reason for this finding is unclear due to limited relevant research. In addition, meta-regression demonstrated that measurement tools and duration were moderators of the effect of exercise interventions on LMS. It implied that the effect of exercise interventions on LMS was influenced by measurement tools and duration, which is consistent with the results of the subgroup analysis.

### 5.3. Object control skills

The meta-analysis demonstrates that exercise intervention has a moderate positive effect on OCS (SMD = 0.79), which is similar to previous results (9). OCS are complex motor skills that reflect coordination, attention, and information integration by all body systems. It is an essential basis for developing special motor skills. MacDonald et al. found that levels of OCS in children with ASD significantly predicted the Severity of autism. Children with lower OCS were more likely to have deficits in social communicative skills (56). The mountain of motor development theory states that early (1–7 years) and middle childhood (7–12 years) are critical periods for the development of FMS. An earlier FMS acquisition can positively affect subsequent growth and life (57). It proved that the development of FMS occurred unnaturally (58), and appropriate instruction and practice were necessary (59). Bo et al. (60) suggest that the poorer development of FMS in children with ASD may be due to a lack of physical activity participation and an optimal environment for them to practice. Exercise interventions create an appropriate environment for motor development and opportunities to practice, allowing for partial compensation for otherwise deficient motor skills. Therefore, suitable environments and practice opportunities provide targeted instruction and guide repetitive practice for children with ASD. It is essential to their FMS improvement and reinforcements (59).

Furthermore, our meta-analysis found moderate and significant heterogeneity between studies (*I*^2^ = 56.9%). The subgroup analysis indicated that aquatic training interventions could improve the ESs (1.72) on OCS and achieve a more significant effect than motor skill and game interventions. Previous studies have demonstrated that aquatic training intervention improves a variety of gross motor outcomes in children with ASD (61–63). The reason for this finding is that aquatic training interventions provide a variety of sensory stimuli through water temperature, weight reduction, and vestibular input. Meanwhile, the properties of water provide postural support and promote relaxation of spastic muscles, allowing a variety of FMS to be performed depending on the individual's skill level (38). A 12-week aquatic training intervention was conducted with three ASD children aged 11–15 years and showed significant improvements in LMS and OCS (16). The increase in OCS was attributed to aquatic training intervention improving the link between perceptual ability, visual-motor integration, and motor skills. In addition, several studies have confirmed the effect of game on the improvement of OCS. It implied that appropriate types of exercise interventions are vital to improving OCS.

### 5.4. Stability skills

The meta-analysis demonstrates that exercise intervention has a moderate positive effect on SS (SMD = 0.73), which is similar to previous results (64). Balance is the ability of an individual to maintain a specific state of the body under dynamic, static, or kinesthetic conditions (65). Balance control involves a complex interplay between information processing, motor planning, and timing and sequencing of muscle movements (66). Therefore, the reasons for SS improvement in children with ASD can be summarized as follows. First, ankle and knee joints endured adequately exercised during exercise interventions. It effectively improves the integration of associated efferent neuromuscular and sensorimotor inputs, which benefits static postural and dynamic postural control (67). Second, the complex exercise environment constantly stimulated their sensory functions, encouraged positive behavioral and strategic choices, and reduced weight shifts (68). Since SS is the fundament for gross motor skills (running, jumping etc.), especially in the earlier school age (7–10 years) (69), it is critical to improving the SS of children with ASD.

Furthermore, our meta-analysis found moderate and significant heterogeneity between studies (*I*^2^ = 32.7%). The subgroup analysis indicated that a session time of ≥60 min could improve the ESs (1.08) on SS and achieve a more significant effect than that of <60 min. Notably, only one in included studies (≥60 min) adopted a session time of 70 min, while others were 60 min. Because most participants had repeated behaviors and hardly focused on themselves (70), too long a session time made them feel bored and neglected to engage in the intervention experiment. Similarly, there is a significant difference among the interventions with different frequencies (one to three times). Although there were no significant differences in intervention duration, some studies suggest that longer exercise intervention duration has a better effect. Zolghadr et al. (71) found that 12 weeks of balance training was better than 6 weeks for children and adolescents with intellectual disabilities. With the extension of the exercise intervention duration, the neuromuscular system of children with ASD adapts to environmental changes due to repeated stimulation, leading to motor ability development and balance skills improvement. Finally, meta-regression showed that measurement tools and exercise session time were moderators of the effect of exercise interventions on SS, implying that the effect of exercise interventions on SS improved with time and was influenced by measurement tools, which is consistent with the results of the subgroup analysis.

### 5.5. Study limitations

There are several limitations to the present review. Firstly, strict inclusion criteria limited the number of included studies and sample size, which may influence the accuracy. Moreover, some effective exercise interventions may be omitted, for only RCTs were included. Secondly, the wide age span makes it hard to achieve the essential moderating variable of age for the optimal exercise intervention period. Thirdly, various measurement tools were adopted to assess FMS, making it hard to calculate the total ESs and leading to difficulties in synthesizing the results. Fourthly, only a few studies provide scientific monitoring of exercise intensity, which limits the interpretation of heterogeneity. Finally, most studies had no long-term follow-up, and whether exercise intervention has a long-term benefit for FMS remains unclear.

## 6. Conclusions

This systematic review and meta-analysis demonstrate that exercise interventions may have moderate to large beneficial effects on FMS in children with ASD, including LMS, OCS, and SS. To better understand the exercise intervention effects on children with ASD, well-designed studies are necessary. Specifically, In terms of LMS, TGMD is most appropriate for LMS measurements, and the effective intervention frequency is 2 times/week. In terms of OCS, TGMD is also most effective for OCS measurements, the intervention frequency of 2 times/week is most efficient, and aquatic training is the optimal type of exercise intervention to enhance OCS. In terms of SS, standardized measurement tools (e.g., Borg scale, Stork test) are most effective for SS measurements, the intervention frequency of 2 times/week is most efficient, and exercise session time ≥60 min is more effective. In addition, the effects of children's age and intervention intensity on the effects of exercise interventions need to be further explored.

## Data availability statement

The original contributions presented in the study are included in the article/Supplementary material, further inquiries can be directed to the corresponding author.

## Author contributions

Y-QJ and HT: acquisition and analysis of data for the study, drafting the paper, and interpretation of data for the study. Y-QJ, Z-YY, and Z-YZ: design and acquisition of data for the study. QY: drafting the paper, revising the paper for important intellectual content, and interpretation of data for the study. All authors contributed to the article and approved the submitted version.
