# Supplementary material for: Effectiveness of exercise intervention on improving fundamental motor skills in children with autism spectrum disorder: a systematic review and meta-analysis
Source: Front Psychiatry. 2023 Jun 12;14:1132074. doi: 10.3389/fpsyt.2023.1132074 (PMC10291092; doi:10.3389/fpsyt.2023.1132074)
Supplement: Supplementary file 1 [file Data_Sheet_1.PDF]

Supplementary Material

Effectiveness of Exercise Intervention on Improving Fundamental Motor Skills in Children with Autism Spectrum Disorder: A Systematic Review and Meta-Analysis

Yu-Qin Ji [1], Hao Tian [2,3], Ze-Yu Zheng [4], Zhuo-Yan Ye [5], and Qiang Ye [2*]

*** Correspondence:** Qiang Ye: yeqiang@nsi.edu.cn

1 Supplementary Figures and Tables

1.1 Supplementary Figures

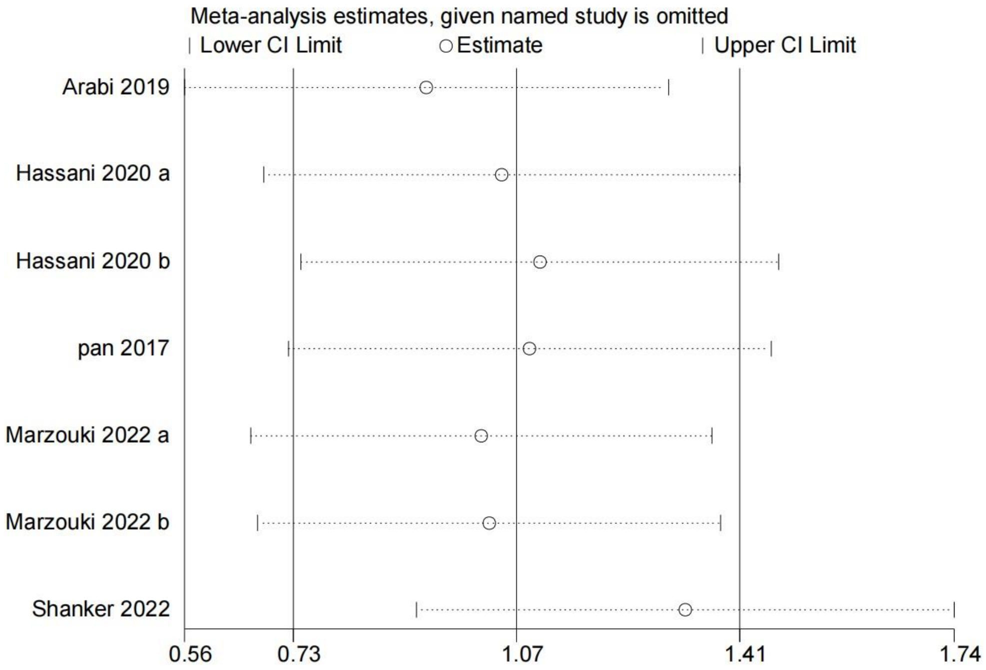

Supplementary Figure 1. sensitivity analysis for LMS

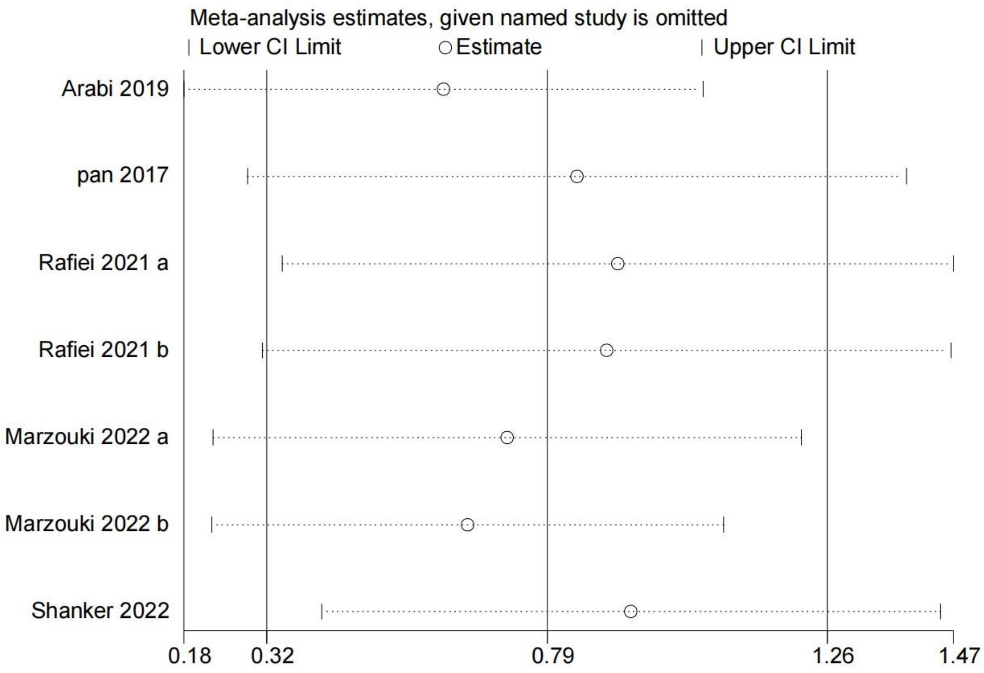

Supplementary Figure 2. sensitivity analysis for OCS

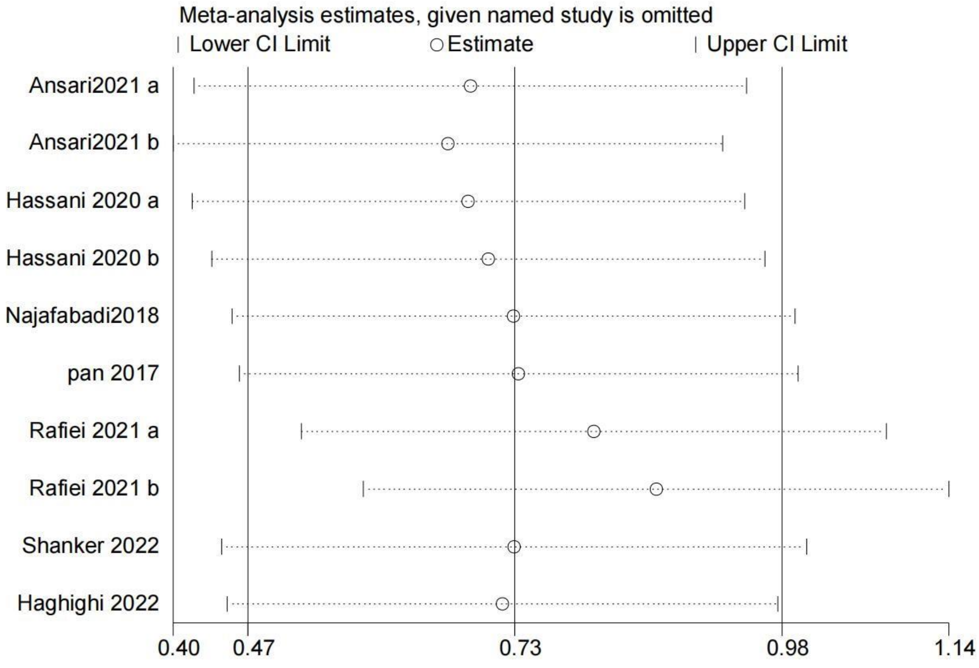

Supplementary Figure 3. sensitivity analysis for SS

1.2 Supplementary Tables

Supplementary Table 1

①	autism OR autistic OR Asperger syndrome OR ASD OR PDD-NOS
②	Child* OR adolescen* OR youth* OR teen*
③	play* OR “physical education*” OR “endurance train*” OR “aerob* train*” OR “weight train*” OR “resistance train*” OR “strength train*” OR “physical fit*” OR “physical activ*” OR exercis* OR sport*
④	FMS OR “motor skill*” OR “movement skill*” OR “motor abilit*” OR “motor competenc*” OR “motor proficienc*” OR “motor development” OR “motor performance” OR “motor function” OR “motor impairment” OR “motor fitness” OR “movement proficien*” OR “locomotor” OR “gross motor” OR “object control” OR “manipulative skill*” OR “manipulative control” OR “object manipulation” OR stability OR balance OR gait* OR postur* OR coordination

Supplementary Table 2. GRADE assessment of the evidence of certainty for exercise intervention effects.

Certainty assessment							№ of patients		Effect		Certainty	Importance
№ of studies	Study design	Risk of bias	Inconsistency	Indirectness	Imprecision	Other considerations	Intervention	Control	Relative (95% CI)	Absolute (95% CI)		
locomotor skills												
5	randomised trials	serious	not serious	not serious	serious	strong association	86	76	-	SMD 1.07 SD higher (0.73 higher to 1.41 higher)	⨁⨁⨁◯ Moderate	IMPORTANT
object control skills												
5	randomised trials	serious	serious	not serious	not serious	none	105	98	-	SMD 0.79 SD higher (0.32 higher to 1.26 higher)	⨁⨁◯◯ Low	IMPORTANT
stability skills												
7	randomised trials	serious	not serious	not serious	serious	none	135	131	-	SMD 0.73 SD higher (0.47 higher to 0.98 higher)	⨁⨁◯◯ Low	IMPORTANT

Supplementary Table 3. Regression analysis for LMS.

ES	No. of studies/comparisons	Coef.(β)	Std. err.	95% Conf. Interval	P
Measurement*	7	-0.996232	0.3827848	-1.980212, -0.0122525	0.048
Type	7	0.064722	0.3186208	-0.7543185, 0.8837631	0.847
Duration*	7	0.996232	0.3827848	0.0122525, 1.980212	0.048
Frequency	7	0.284400	0.3019606	-0.4918142, 1.060615	0.390
Time	7	-0.172117	0.4714987	-1.384144, 1.039908	0.730

*shows that the data differ. *$p < 0.05$

Supplementary Table 4. Regression analysis for OCS.

ES	No. of studies/comparisons	Coef.(β)	Std. err.	95% Conf. Interval	P
Measurement	7	-0.569859	0.2270092	-1.153405, 0.0136864	0.054
Type	7	0.345742	0.3358778	-0.517659, 1.209144	0.351
Duration	7	0.629188	0.5265861	-0.7244443, 1.982821	0.286
Frequency	7	0.088981	0.3638452	-0.8463124, 1.024275	0.817
Time	7	-0.440078	0.5836194	-1.94032, 1.060163	0.485

Supplementary Table 5. Regression analysis for SS.

ES	No. of studies/comparisons	Coef.(β)	Std. err.	95% Conf. Interval	P
Measurement*	10	0.581979	0.1850887	0.1551636, 1.008794	0.014
Type	10	0.372284	0.2353618	-0.170461, 0.9150295	0.152
Duration	10	-0.172136	0.3360524	-0.9470738, 0.6028029	0.622
Frequency	10	-0.263797	0.1566112	-0.6249426, 0.0973495	0.131
Time*	10	-0.695826	0.2592773	-1.29372, -0.097931	0.028

*shows that the data differ. *$p < 0.05$
